# Planning a digital intervention for adolescents with asthma (BREATHE4T): A theory‐, evidence‐ and Person‐Based Approach to identify key behavioural issues

**DOI:** 10.1002/ppul.26099

**Published:** 2022-09-06

**Authors:** Stephanie Easton, Ben Ainsworth, Mike Thomas, Sue Latter, Rebecca Knibb, Amber Cook, Sam Wilding, Michael Bahrami‐Hessari, Erika Kennington, Denise Gibson, Hannah Wilkins, Lucy Yardley, Graham Roberts

**Affiliations:** ^1^ NIHR Southampton Biomedical Research Centre, University Hospital Southampton NHS Foundation Trust Southampton UK; ^2^ Human Development and Health, Faculty of Medicine University of Southampton Southampton UK; ^3^ Department of Psychology, Faculty of Humanities and Social Sciences University of Bath Bath UK; ^4^ Primary Care and Population Sciences, Faculty of Medicine University of Southampton Southampton UK; ^5^ School of Health Sciences University of Southampton Southampton UK; ^6^ School of Psychology, College of Health and Life Sciences University of Aston Birmingham UK; ^7^ Clinical Trials Unit, University Hospital Southampton Southampton UK; ^8^ Asthma+Lung UK London UK; ^9^ Physiotherapy Department University Hospital Southampton Southampton UK; ^10^ Centre for Clinical and Community Applications of Health Psychology University of Southampton Southampton UK

**Keywords:** adolescence, asthma, breathing retraining, digital intervention, self‐management

## Abstract

**Objectives:**

To describe a transparent approach to planning a digital intervention for adolescents to self‐manage their asthma using breathing retraining (BRT), based on an existing, effective adult intervention (BREATHE).

**Methods:**

A theory‐, evidence‐, and Person‐Based Approach was used to maximise the effectiveness and persuasiveness of the intervention. A scoping review and semistructured interviews with target intervention users (*N* = 18, adolescents aged 12−17 years with asthma and parents) were carried out to explore user perspectives, barriers, and facilitators towards the intended behaviours and potential intervention features. The combined evidence was used alongside and to inform theory‐based activities and enabled iterative planning of the intervention.

**Results:**

The scoping review identified themes relating to user‐specific self‐management issues, content, education, training needs, and features for a digital intervention. Interviews elicited potential barriers to intended behaviours such as the anticipated embarrassment of using BRT and concerns around remaining calm. Facilitators included BRT delivered by adolescents who share experiences of asthma and information for performing exercises discreetly. Relevant theoretical frameworks ensured that appropriate psychological constructs were targeted. A behavioural analysis identified six intervention functions and thirty behaviour change techniques. Logic modelling mapped the programme theory and mechanisms, which aims to improve adolescent asthma‐related quality of life.

**Conclusions:**

This study gives a transparent insight into the approach followed to plan a self‐guided BRT intervention for adolescents and has led to identification of key behavioural issues, enabling relevant intervention content to be chosen. Insight has been given into adolescent perceptions of BRT, which facilitated development of the prototype intervention.

## 1 BACKGROUND

Asthma is an intermittent, long‐term disorder characterised by wheeze. It affects over 339 million people globally and is the most common noncommunicable disease in children and adolescents,^1^, ^2^ with 1 in 14 adolescents having asthma.^3^ Adolescence is recognised as a challenging period of rapid change and brain development.^4^ During this period adolescents tend to be heavily influenced by their peers and are more likely to take health‐related risks, including experimenting with drugs, alcohol, and cigarettes.^5^, ^6^ These physical, behavioural, and psychological adjustments can have an impact on the management of long‐term conditions. For adolescents with asthma, despite the availability of effective medications, engagement with treatment is often suboptimal and this age group tend to have poor asthma outcomes, including high mortality rates, and particularly poor quality of life.^7^, ^8^, ^9^ In addition, adolescence presents a time of transition to adulthood as disease management shifts from parents and carers and requires greater adolescent responsibility and self‐management.^10^ These unique factors mean that adolescent patient needs differ from adults, and thus approaches to treatments should be tailored accordingly.

An increasingly popular, adjuvant approach to asthma pharmacotherapy is breathing retraining (BRT), which is recommended to manage symptoms and to improve quality of life.^11^ The prevalence of dysfunctional breathing in children is currently unknown, however one study suggests it may be experienced by as many as 55% of children and adolescents with asthma.^12^, ^13^ It is sometimes known as hyperventilation, vocal cord dysfunction or inducible laryngeal obstruction.^12^, ^14^, ^15^, ^16^ It is characterised by increased respiratory rate, the inappropriate use of accessory muscles and paradoxical vocal cord movement.^12^, ^16^ Dysfunctional breathing can exacerbate asthma symptoms, further impact quality of life and increase anxiety.^11^, ^12^ Young adults with asthma often report anxiety and panic alongside asthma symptoms, in particular if medication is not within reach.^17^ Both adolescents and parents recognise the importance of staying calm and reducing panic to help control breathing.^17^

Breathing exercises are traditionally taught by a physiotherapist and provide techniques to increase breathing efficiency, control and relaxation. This can be costly and requires the availability of a qualified professional. Bruton et al.^18^ developed a successful and cost‐effective digital intervention for self‐guided BRT in adults (BREATHE). A randomised, controlled trial demonstrated that the home‐based digital video disc and booklet programme was as effective in improving quality of life as face‐to‐face therapy, in comparison to usual care.^18^ Despite these promising preliminary observations, systematic reviews have identified a lack of similar intervention studies exploring the effectiveness of BRT in younger patients.^19^, ^20^, ^21^, ^22^ Yet, a similar, digital, self‐management approach is likely to be useful for this age group.^9^, ^23^, ^24^ Initial research and work with patient and public involvement (PPI) demonstrated that the adult BREATHE intervention needs repurposing and optimising to engage younger patients.

Strategic planning of complex interventions is important and increases their chance of success.^25^ The UK Medical Research Council (MRC) have recently extended their guidance to support the planning, development, feasibility, and evaluation of complex interventions, emphasising overarching considerations such as context and stakeholder involvement.^26^, ^27^ As the planning and development stage preceding a feasibility trial (Clinical trials; [NCT05006703]), this paper describes the process to plan and repurpose the adult BREATHE intervention into a BREATHE4T adolescent intervention. The aim was to codevelop a draft, prototype behavioural, self‐management, digital intervention with adolescents with asthma. We followed the Person‐Based Approach, which involves a systematic theory‐, evidence‐, and Person‐Based Approach to development, in line with MRC guidance.^26^, ^27^, ^28^ It has been used to develop several effective and cost‐effective behavioural interventions to help manage various long‐term health conditions.^18^, ^28^, ^29^, ^30^, ^31^, ^32^ The iterative nature of the approach allows changes to be made throughout planning and development to maximise the intervention's persuasiveness, relevance and potential to change behaviour, and thus leading to improved overall health outcomes.^28^, ^33^

## 2 METHODS AND RESULTS

### 2.1 Approach

A theory‐, evidence‐, and Person‐Based Approach was used to plan, repurpose and develop an existing adult asthma intervention to be suitable and engaging for adolescents.^28^

**Figure 1:**
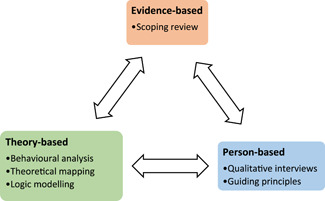
An overview of the key elements involved in planning the Breathe4T intervention [Color figure can be viewed at wileyonlinelibrary.com]

The systematic approach to planning is presented in Figure 1. Detailed intervention planning included three parts: (1) a rapid scoping review (evidence‐based), (2) an in‐depth qualitative interview study (person‐based) and (3) behavioural analysis, logic modelling and theoretical mapping (theory‐based). This combined iterative process enabled a deep insight into the perspective and experiences of the target population.^33^ The scoping review and qualitative interviews were combined to identify the key, context‐specific, behavioural issues, and needs that an adolescent BRT intervention needs to address. In line with the Person‐Based Approach, these methods and the other planning activities were conducted in parallel and combined to iteratively create a draft intervention prototype. This also enabled a set of guiding principles to be generated, which outline the key design objectives and key intervention features relevant to this intervention. The following behaviours are intended to be targeted by the intervention: (1) to practice BRT, (2) to be able to identify when to use breathing exercises (self‐manage), (3) to implement breathing exercises and (4) to engage with the intervention.

Key stakeholders were involved in decision‐making throughout, including adolescent asthma patients, and parents. Monthly development meetings were held with the core project team which consisted of clinical, psychological, and behavioural expertise, plus support from Asthma UK. These meetings predominately aimed to keep the project focused and to resolve challenges.

#### 2.1.1 PPI

Two parallel panels of six adolescents with asthma and five parents were recruited at the start of the study and met with the study team quarterly. In‐between meetings, regular contact was made with the adolescents via their preferred choice of communication (a social media app and WhatsApp) whilst parents communicated more traditionally via email. PPI members actively contributed in various ways by inputting to the study design, documents and recruitment activities and providing feedback on the intervention prototype and language to ensure suitability for an adolescent audience.

### 2.2 Rapid scoping review (evidence)

#### 2.2.1 Purpose

To identify relevant barriers, facilitators, and contextual issues that may influence engagement with a digital, behaviour change intervention for adolescents with asthma from existing evidence.

#### 2.2.2 Methods

In keeping with development timeframes, a rapid scoping review was conducted to collate existing evidence. We followed Arskey and O'Malley's five stage framework.^34^ The team's previous research had identified evidence of barriers and facilitators toward adolescent asthma self‐management and were included within the review.^9^, ^17^, ^23^, ^35^ Three further questions were identified as evidence gaps by the core study team and were used to focus the searches: (1) what are the barriers and facilitators toward adolescent engagement with digital self‐management interventions? (2) is BRT effective as an adolescent asthma intervention? and (3) what makes an effective adolescent peer‐led intervention? Searches were conducted in Medline (Ovid), Embase, Psycinfo and Cinahl. Additional studies were identified through reference lists of included papers. Endnote software was used to manage references and to remove duplicates. Data were extracted on authors, methodology, intervention type, and key findings (Supporting Information: E‐Table 1). In line with similar studies, thematic analysis was conducted on the extracted data. Potential barriers and facilitators that may influence engagement with the intervention were identified from the three areas above and organised into a summary table of key findings (Table 1).^61^

**Table 1:** Summary of key barriers and facilitators identified in the scoping review

Theme	Barriers	Facilitators
User‐specific self‐management issues	Forgetfulness^9^, ^17^, ^36^, ^37^, ^38^, ^39^, ^40^, ^41^, ^42^ Competing demands/priorities^9^, ^36^, ^37^, ^38^, ^39^ Busy or chaotic home life^20^ Too many reminders—“annoying”^42^ Embarrassment or drawing unwanted attention to asthma^9^, ^17^, ^35^ Reliance on parents^9^, ^17^ Belief that already doing a good job of managing asthma^36^ Lack of motivation^9^, ^17^, ^43^ Negative beliefs, attitudes or perceptions towards asthma^9^ Lack of social support and poor communication^9^, ^17^, ^35^ Greater anxiety^35^	Routines and cues^9^, ^17^, ^20^ Appointment/medication reminders^9^, ^17^, ^36^, ^37^, ^38^, ^39^, ^40^, ^41^, ^42^, ^43^, ^44^, ^45^ Able to customise or schedule reminders^38^, ^40^, ^42^ Support from peers, parents and caregivers^23^, ^37^, ^42^, ^46^ Acceptance of having asthma^36^, ^39^, ^42^ Taking responsibility (and having the confidence to)^17^ Achieving or maintaining normalcy^37^, ^38^, ^39^, ^43^ Feeling less limits in daily activities^36^, ^37^, ^38^, ^39^, ^43^ High self‐efficacy^23^ Feeling in control of asthma symptoms^40^ Often have a smartphone available^37^ Goal setting^39^, ^43^, ^47^ Avoiding hospitalisation^48^
Content, education and training needs	Lack of or inadequate asthma knowledge^17^, ^36^, ^38^ Not having enough information or understanding about the condition, symptoms, triggers, severity and risks^23^, ^36^, ^38^, ^40^, ^41^, ^42^, ^43^, ^45^, ^49^ Unrelatable peer mentors^50^ Difficulties communicating with HCPs^9^, ^17^, ^48^, ^51^ Prefer not to replace advice from HCPs (supplement)^44^, ^49^	Being given new ways to control asthma^17^ Greater understanding of consequences of the condition^23^ Having control over symptoms^36^, ^38^, ^39^, ^42^ Interacting/hearing experiences from others with asthma^36^, ^37^, ^38^, ^42^, ^43^, ^44^, ^46^, ^48^, ^52^, ^53^ Prefer advice from peers (particularly older)/change is more likely to occur is someone relatable delivers the message^52^, ^54^, ^55^ Prefer to learn from demographically similar other^56^ Credible/trusted source of information^38^, ^44^, ^45^ Reduce need for appointments with HCPs^49^ Prefer to learn breathing exercises along to a CD (than alone)^20^, ^57^ Using breathing exercises to relax, feel calm and fall asleep more easily at night^57^ Encouragement to contact HCPs for further information^44^
Features of digital interventions	Information cluttered/clumped^43^, ^45^ Outdated app design^42^ Too many features^45^ Issues with login processes^42^, ^58^ Difficulty understanding how to use an app/moving between screens^43^, ^45^ Rewards that don't translate into anything^41^ Poor accessibility (WiFi, devices etc.)^42^, ^45^	Clean, professional and organised^41^ Use of visual aids such as colour, pictures, graphs and charts^41^, ^43^, ^45^ Video tutorials/picture explanations to aid understanding and teach new skills^36^, ^37^, ^40^, ^42^, ^43^, ^45^, ^48^, ^59^ Ease of use^38^, ^40^, ^42^, ^43^, ^45^, ^47^, ^48^, ^52^ Tracking and monitoring (symptoms and triggers)^36^, ^38^, ^40^, ^43^, ^45^ Customisable/tailored/personalised information^37^, ^42^, ^44^, ^45^, ^59^, ^60^ Able to share information^36^, ^37^, ^41^, ^42^, ^47^, ^49^ Rewards for adherence^51^ Inspirational/motivational messages^42^

Abbreviation: CD, compact disc; HCPS, health care professionals.

#### 2.2.3 Results

Thirty‐five papers were included in the review. Three key themes were constructed from the data: (1) user‐specific self‐management issues (2) content, education, and training needs and (3) features of digital interventions. Barriers and facilitators were each labelled according to the search they were identified in. A full table of findings is provided in Supporting Information: E‐Table 2.

### 2.3 Qualitative interviews (person)

#### 2.3.1 Purpose

To elicit user views of potential intervention content and toward target behaviours, whilst exploring any further potential barriers, facilitators, or behavioural challenges that the intervention may need to address.

#### 2.3.2 Methods

Ethical approval was obtained from an NHS ethics committee (19/YH/0338). Participants were purposefully recruited from a secondary care asthma clinic. Eligible participants were between 12 and 17 years old with physician‐diagnosed asthma, or their parent/guardian. Participants were excluded if they had coexisting respiratory conditions or were an existing PPI member. Potential participants and their parents (*N* = 68) who had an upcoming clinic appointment were sent information about the study and 18 (9 parent and teenager dyads) chose to take part. All participants gave fully informed consent and completed a demographic questionnaire before the interview (Table 2). Semistructured topic guides (Supporting Information: E‐Text 1) focused on target behaviours (breathing exercises) and potential intervention features (such as videos or rewards). As a prompt, participants were shown an example of the existing intervention for adults (www.breathestudy.co.uk) and asked their likes, dislikes and to suggest any improvements they would make. Interviews were recorded and transcribed verbatim. All transcripts were anonymised, and names replaced with pseudonyms. Repeated readings of transcripts and listening to the recordings ensured familiarisation with the data. Inductive, thematic analysis was used to identify views of target behaviours and any adolescent‐specific behavioural challenges.^62^ Key issues and perceptions of potential intervention features (both positive and negative) were identified.

**Table 2:** Adolescent interview participants' self‐reported demographic information

Participant demographics	*N* (%)
Total (adolescents)	9
Gender	
Female	5 (55%)
Age, years	
12−13	5 (55%)
14−15	1 (11%)
16−17	3 (33%)
Ethnicity	
White British	8 (88%)
Self‐reported asthma triggers	
Weather	5 (55%)
Pollen	6 (66%)
Dust	7 (77%)
Pets	8 (88%)
Colds	7 (77%)
Cigarette smoke	4 (44%)
Exercise	9 (100%)
Professionals seen about asthma	
GP	7 (77%)
Primary care nurse	4 (44%)
Hospital consultant	9 (100%)
Missed preventer	
Never	3 (33%)
Occasionally	3 (33%)
Once a week	2 (22%)
Half the time	1 (11%)
One or more steroids courses needed in the last year	4 (44%)
>2 days of school missed in last year	6 (66%)
Ever admitted to hospital because of asthma	5 (55%)
Has eczema	3 (33%)
Has hay fever	7 (77%)
Food allergies	7 (77%)
Nonsmoking family	9 (100%)

Most interviews took place in a hospital research setting (*n* = 11) or, where participants preferred, their home (*n* = 4) or via the telephone (*n* = 3). Interviews were conducted by a research assistant (SE), and lasted an average of 32 min (range 16−56 min).

#### 2.3.3 Findings

Key findings arising from the adolescent and parent interviews are described below. Example quotes are presented in Table 3. Three main themes were identified: views towards practicing and using breathing exercises, preferences for the intervention features and preferences for delivering BRT. Participants described various issues around anticipated embarrassment, particuarly within certain environments and this was identified as its own subtheme within practicing and using breathing exercises.

**Table 3:** Key barriers and facilitators arising from qualitative interviews with adolescents with asthma and their parents

Barrier/facilitator	Participant quotes
Theme 1: Views toward practising and using breathing exercises	
*Adolescents felt positive about trying breathing exercises.*	“I think it's a good idea because when you are having an asthma attack if you start to breathe faster it makes your chest even worse and then, so if you start to slow down your breathing then it does help you when you're having an asthma attack” (P17, F, 17 years)
Some participants (adolescents and parents) described possible time constraints towards practising breathing retraining.	“If you're doing it for a long time, over a long period of time every time you have to go on it then you wouldn't want to do it if it takes half an hour” (P5, F, 13 years) “She does play some sport as well, she's in the first team for hockey for her year and she used to ride but she's given that up lately because there has just been no time and what else does she do? She's started playing netball as well and she plays the drums” (P8, mother of 13 year old female)
A few adolescents had experienced discomfort after using breathing exercises previously.	“I felt it put quite an unnecessary strain on your diaphragm and your upper shoulders coz I have a problem with breathing up here [hand on chest], rather than in my stomach so I felt if you had a bit of difficulty a bit later on in the day after doing these breathing exercises, it would put quite a bad strain, you'd feel quite a lot more pain than you would if you didn't do the breathing exercises” (P3, M, 16 years)
*Some adolescents felt breathing exercises may also benefit those without asthma.*	“Breathing exercises are always going to be a good thing even if you don't have asthma, it's still going to be good…anybody that has a lack of breath it will be useful for” (P13, M, 12 years)
Adolescents and parents described a possible lack of motivation to practice breathing exercises.	“I wasn't even doing them once a day, I was doing it whenever I saw the physiotherapist so it was quite infrequent, really bad, yeah, I said I would do it but I never did [laughs]” (P3, M, 16 years) “He's a teenager, he's lazy isn't he? If he's got coursework to do, he's got to do that hasn't he?” (P4, mother of 16 year old male) “The most difficult group to reach I would think are boys…they won't bother, it's as simple as that” (P8, mother of 13 year old female)
Adolescents suggested their environment or location may impact their ability to use breathing exercises.	“I think in certain lessons or if you're with a group of people then you can't always get a moment alone to just relax” (P11, F, 16 years)
Some parents had concerns about their adolescent's ability to calm down during an asthma attack.	“I think if you're in the throes of an asthma attack it's so frightening that I think it would take quite a lot of presence of mind to talk yourself into calming down and doing that and so, maybe the more mature, but not exclusively, but teenagers can actually be more needy than 5 year olds at times…” (P12, mother of 16 year old female)
*Parents discussed the long‐term benefits as a possible way of increasing adolescent's motivation towards using breathing exercises.*	“With this…the long term benefits I'm guessing would be that they learn the exercises, they would be able to do more of what they want to do, do more of the PE and more of the Judo, so I guess telling him, if he learns them and pays attention to them then he might be able to do more things. He might be able to take less medicine because he can control his breathing, I mean that might work with him” (P2, mother of 15 year old male)
Some parents were concerned that their teenagers may lack support at school.	“Also just the school environment is difficult because actually an awful lot of teachers don't realise how critical asthma can be” (P12, mother of 16 year old female) “I think the teacher is the barrier…it's actually to be able to have the confidence to go to the teacher and say I think this is about to happen, I need to step out…” (P14, mother of a 12 year old male)
Some adolescents felt an inhaler could relieve panic more quickly than taking the time to use breathing exercises.	“I really find it difficult…immediately the first thing you do is panic, so you reach for that blue inhaler and you need to take it there and then really, because that was my strategy and it still is but I could never calm myself down in that situation and then to focus on the breathing exercises…so I think go for the blue inhaler first and then once it's taken the edge off, off the asthma then start going into breathing exercises” (P1, M, 15 years)
Subtheme: Anticipated embarrassment or self‐consciousness	
Adolescents and parents anticipated feelings of self‐consciousness and embarrassment in public.	“I'd have just been a bit nervous and I don't like doing stuff that brings attention to me so it would have just made me anxious and not want to do it” (P1, M, 15 years) “They get embarrassed, don't they? So I know Josie gets embarrassed using her inhaler at school so she has to go and use it in the toilet, so she'd probably be embarrassed doing it in front of people, she wouldn't be embarrassed at home but she would be embarrassed about doing it at home if she didn't have a place to do it on her own…” (P16, mother of 13 year old female) “She does say she feels embarrassed or awkward when she uses her inhalers in public and she gets teased by other children for it” (P8, mother of 13 year old female)
Adolescents and parents had mixed views on the idea of using breathing exercises around friends, especially those without asthma.	“Well because none of my friends have asthma, I don't think they would be used to it because they don't know anyone with asthma or in their family and I think I'm the only one they know with asthma…I think they just wouldn't really know what I'm doing and think, maybe think it's a bit weird and it will be a bit emb‐, a bit uncomfortable or something” (P15, F, 13 years) “I also have some other friends with asthma and we all help each other out” (P13, M, 12 years) “I don't know that peer pressure is so much of an issue now to sort of making teenagers feel awkward about doing something because actually there much, they are very supportive of each other” (P12, mother of 16 year old female)
*Adolescents and parents discussed knowing how the exercises could be performed discreetly to avoid embarrassment.*	“Just teaching the exercises in a way that you wouldn't have to be so obvious about it so in the video they wanted you to lie down on a bed and relax your shoulders and stuff but that's I guess that's harder to do” (P17, F, 17 years) “I don't think she would do it out and about, I mean if she could do it without being noticed then maybe she would” (P18, mother of 17 year old female)
*A few adolescents described coping strategies that may mitigate against their feelings of embarrassment.*	“If I needed to breathe and it wasn't subtle I would do it, but I don't think it's that noticeable anyway because when you are breathing, I mean people don't really look at your stomach to see how far out it is going and they don't really look at you to see how much you are breathing but if they did ask, like if you're not talking and breathing instead then I'd just say” (P5, F, 13 years)
**Theme 2: Preferences for the intervention features**	
*Several adolescents and parents suggested an app would be a preferred format.*	“It would be good if it was an app, if it was an app on your phone or something then you could always have a look on it then they could get a notification when you went on” (P5, F, 13 years) “It would be good if there was an app on her phone so if she was feeling unwell at school then she could do, follow the stuff on an app on her phone because that's what they all do now isn't it with their apps and stuff?” (P16, mother of a 13 year old female)
Adolescents and parents suggested users wouldn't want to read too much writing*/and had a preference to view visual content.*	“If there's just long bits of text then you probably aren't going to read it and it's just, yeah boring to read whereas if it's just a quick video or quick pictures or something then it's a lot easier to access and more interesting and so you are more likely to actually do it” (P17, F, 17 years) “They are not readers these days kids, they are just not readers. There are very few and far between that are” (P8, mother of 13 year old female)
*Generally, participants felt a website needed to be professional, organised and easy to use.*	“I think it just needs professionalism and it needs to look appealing to people my age, sort of thing” (P3, M, 16 years) “If it's easy to understand, so it's not muddled up and everything is easy to access and just colours really, if it looks nice, if it's old and uninteresting then people won't want to use it” (P1, M, 15 years)
*Reminders and notifications were viewed positively by participants, if not sent too frequently.*	“I think notifications are useful but I think you have sort of periodically, I always get annoyed at apps or like websites when it notifies me once an hour or something, just sort of spreading it out slightly so it's not always nagging you, it's just gently reminding” (P11, F, 16 years)
There were mixed views on the use of rewards by both adolescents and parents.	“Well I probably wouldn't need a reward for just looking at a website or something, I'd just do it because it will help” (P9, M, 14 years) “Rewards, it makes you want to do more and earn another, it entices you to keep going and achieving.” (P1, M, 15 years) “Any reward for kids is good…book tokens, earning stuff for crafts stuff that she's interested in, I don't know, but anything they can get something out it” (P16, mother of 13 year old female) “I think for this, the main thing is the benefits, I mean to be able to go out with his friends and be a normal teenager” (P2, mother of 15 year old male)
*Adolescents liked the idea of an animation to breathe in time with.*	“It's clear what you need to do, you need to breathe in for a bit and then out…I like that one because it's also engaging, you can just watch it go around and it will calm you down whilst you're watching it and relax you” (P15, F, 13 years)
There were mixed views on parental involvement. Some parents felt their teenagers should start taking responsibility for their asthma, whilst others still wanted to support them.	“I think them having access to see what's on the website but I think if they were getting notifications of what I was doing then I would kind of get annoyed if they started nagging me or I think anyone my age would get annoyed with that” (P13, M, 12 years) “Hearing from parents helps because they have lived longer than you and have good advice that you can, a lot of people can use…if they realise I'm doing something wrong, then they can correct me which helps” (P7, F, 13 years) “I'd like to know what he's doing and how to do the exercises to check he is doing them properly” (P2, mother of 15 year old male) “I think he's transitioning into adulthood now, he needs to take responsibility for it” (P4, mother of 16 year old male)
*Progress monitoring was viewed positively as an intervention feature.*	“Some kids do like to chart their progress and to see that they are getting towards an end goal” (P8, mother of 13 year old female)
Theme 3: Preferences for delivering breathing retraining	
*Some adolescents expressed interest in understanding why breathing exercises may help, though parents felt adolescents may vary in the level of detail they'd like to know.*	“If you see something happening and you don't know what's happening then you can't really help it but if you see something happening and you know what's happening then you can help it” (P13, M, 12 years) “I think it would be good to know what you're looking at. Even if it's too technical for some, if it's there, some kids are more into it than others, some couldn't care less if they had whatever in their lungs, but I think someone like Aaron, he would like to see that” (P4, mother of 16 year old male)
*All participants felt demonstrations of breathing exercises would be more relatable by a young person that understands or has experienced asthma.*	“Because I'm also a teenager and if I was to watch a teenager doing it then I would definitely know it works but if I was watching an adult then I could think well it might work for adults but not for kids” (P15, F, 13 years) “I think because that is the world they access now, they learn how to put their make up on looking at other teenagers putting their make up on… it's just their world now, they tend to listen to people on the screen far more than they do anybody else…if another teen was showing it and telling them why and how it benefits and everything else then I think that would, she's most likely to take notice of that” (P18, mother of 17 year old female)
*A few participants felt step‐by‐step instructions would be useful.*	“If there were pictures but they had steps under them then that would be good…maybe because if you were out and you could feel your chest being tight and you wanted to like go through the breathing exercise, you can't really watch the video but you could just go through the steps so that would be good” (P17, F, 17 years)
Both adolescents and parents felt the adult intervention videos were too clinical and suggested they'd prefer to see more relatable settings.	“Somewhere where you can get your audience's attention, like possibly not a doctor's office, it's a little bit boring” (P1, M, 15 years) “Maybe in somebody's house or something like that in a proper bed because when you are at home is when you would feel the most safe than when you are somewhere else” (P15, F, 13 years) “I don't like the clinical setting, I suppose most of the videos now that seem to appeal are just young people just talking in their bedrooms, aren't they? Just a normal everyday place, in the kitchen at home in the bedroom, down the park, just not a proper set up if you like?” (P18, mother of 17 year old female)
*Participants felt videos should be kept short.*	“I'd rather just sort of like have her say ok we're doing this and then start showing it instead of the whole talking beforehand” (P11, F, 16 years)

*Note*: Facilitators are italicised, barriers are in non‐italic font.

##### Views towards practicing and using breathing exercises

Overall, both adolescents and their parents were positive about a BRT intervention. Some barriers were raised toward using the breathing exercises including time constraints, parental concerns in the ability to calm down when experiencing asthma symptoms and potential feelings of discomfort. In addition, a key issue discussed by almost all adolescents and parents were potential feelings of embarrassment or self‐consciousness that may influence their ability or motivation to carry out breathing exercises. Some participants felt they'd be supported by friends or particuarly others with asthma, but a few felt they'd still be too embarrassed. Adolescents and parents felt that being able to perform the exercises discreetly or in private would be a facilitator.

##### Preferences for intervention features

Participants described a preference for an intervention that is easy to use, informative and visual. When shown the adult intervention, most participants felt it had too much text and both adolescents and their parents' suggested preferences for more colour, videos, and pictures. In line with the literature, both adolescents and their parents described a challenge around forgetfulness and therefore felt reminders would be a useful and important feature of an intervention for this age group. Some participants felt they would get annoyed if they received them too often and one described wanting gentle reminders. Other features viewed positively included progress charts and breathing animations. There were mixed views on the use of rewards as some participants felt they were unnecessary and that asthma improvements would be the biggest benefit, though others suggested they might be motivated by reward systems and particuarly if it led to a physical reward, such as gift tokens.

##### Preferences for delivering BRT

Participants suggested breathing techniques would be best demonstrated by someone closer in age to help them to connect to the examples and to recognise the exercises can work for them. Some adolescents and their parents also suggested they'd have an interest in understanding the rationale for how breathing exercises work to understand how they might help. Though, some parents suggested adolescents may require varying levels of detail. Other facilitators included the techniques being demonstrated in relatable environments such as at home or in a sports field, as opposed to a doctor's office and the use of step‐by‐step instructions.

### 2.4 Behavioural analysis (theory)

#### 2.4.1 Purpose

To systematically identify and describe intervention components using behaviour change theories (behaviour change wheel [BCW],^63^ behaviour change techniques taxonomy [BCTv1]^64^ and theoretical domains framework [TDF]^65^), mapped onto evidence identified in the scoping review and qualitative interviews.

#### 2.4.2 Methods

Behaviour change theory uses a common language to allow comparison of behavioural techniques between interventions.^63^ The BCW is a theoretical framework that synthesises techniques found in the research literature.^63^ The COM‐B model suggests that at least one of the following factors must be present for behaviour change to occur; capability, opportunity, and motivation.^63^ To undertake a behavioural analysis, the intervention's four target behaviours were specified and recorded in a table; (1) practice breathing exercises, (2) identify when to use breathing exercises (self‐management), (3) implement breathing exercises and (4) effectively engage with the intervention. Potential barriers and facilitators were identified for each behaviour based on findings from the qualitative data (scoping review and interviews) and stakeholder input. Appropriate intervention components designed to elicit behaviour change were selected to address each barrier and facilitator in correspondence with the target behaviours. Each component was mapped onto the BCW to identify the constructs (e.g., physical capability) that needed to be targeted for the desired change to be achieved, in addition to the intervention functions (e.g., training) that would allow this to change. The BCTv1, consisting of 93 behaviour change techniques (BCTs), was used to code each intervention component and to ensure that all relevant intervention functions had been utilised and no potentially useful techniques had been missed. Finally, each component was mapped onto the TDF, which consists of 14 domains used to combine theoretical constructs.^65^

#### 2.4.3 Results

The behavioural analysis consisted of 14 pages. The analysis proposes that the intervention will target all six of the COM‐B model components (physical capability, psychological capability, physical opportunity, social opportunity, reflective motivation, and automatic motivation) and will utilise six of the nine possible intervention functions: education, persuasion, training, modelling, environmental restructuring, and enablement. Thirty BCTs are utilised in the Breathe4T intervention.

### 2.5 Logic modelling (theory)

#### 2.5.1 Purpose

To map intervention mechanisms of action to ensure they are appropriately targeted by intervention components.

#### 2.5.2 Methods

In line with the MRC recommendations, a logic model was created to visually map the intervention's programme theory.^26^, ^27^, ^66^ The model combines the findings from the scoping review, behavioural analysis, and qualitative interviews to describe the underlying mechanisms of action, the intervention components expected to contribute to behaviour change and the expected outcomes.

#### 2.5.3 Results

**Figure 2:**
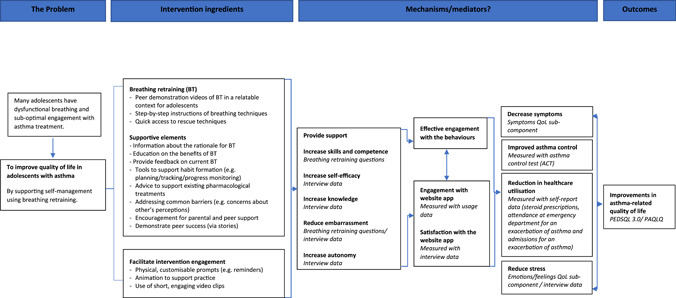
An overview of the logic model for the Breathe4T intervention [Color figure can be viewed at wileyonlinelibrary.com]

An overview of the Breathe4T logic model is shown in Figure 2. The intervention ingredients include the main intervention components that were originally identified during the behavioural analysis included to influence intervention engagement and to target the behavioural constructs that are intended to lead to behaviour change. The logic model displays the theoretical process of intervention features and underlying mechanisms that are intended to lead to the improvement in asthma‐related quality of life, such as increasing skills, competence, and self‐efficacy and reducing embarrassment. A measurement is provided for each mechanism, which will later be considered during a future process analysis. The underlying mechanisms are expected to lead to a decrease in symptoms, improvements in asthma control, a reduction in healthcare utilisation, reductions in stress and therefore result in overall improvements in quality of life (measured by PedsQL^67^ and PAQLQ^68^).

### 2.6 Theoretical mapping (theory)

#### 2.6.1 Purpose

To consider underlying theories to ensure relevant behavioural mechanisms are targeted.

#### 2.6.2 Methods

Self‐determination theory (SDT) comprises of three basic needs considered to influence an individual's intrinsic motivation to carry out a behaviour; autonomy (a desire to be in control of one's own destiny), relatedness (a desire to connect or interact with others) and competency (a desire to be able to achieve a goal).^69^ SDT is particularly relevant for adolescents who may typically lack motivation, confidence or the skills to engage in asthma self‐management.^9^, ^17^ We therefore identified these determinants of motivation when developing the intervention, ensuring that intervention ingredients targeted these constructs as mechanisms. In addition, Rottman's value‐expectancy cognitive framework assumes that a patient's beliefs, based on their own experiences with their medication, may in turn lead to cyclic pattern of nonadherence.^70^ We also ensured ingredients aimed to target these mechanisms, for example by increasing knowledge and encouraging self‐monitoring, therefore leading to more effective behaviour change.

#### 2.6.3 Results

As represented in the logic model (Figure 2), intervention content was mapped on to SDT to ensure the theoretical constructs were targeted. For example, demonstration videos were designed to be peer‐led to increase competency, but also whilst ensuring the intervention would be relatable for adolescents and enabling them to feel supported or to reduce feelings of embarrassment. Reminders, planning, and self‐monitoring tools were included in the intervention to allow participants to enter their own information, scheduling, and choice of modality aiming to increase autonomy. In line with Rottman's theory, users are able to use a progress chart to self‐monitor their inhaler use over time and to analyse their own patterns of behaviours in a diary section.

### 2.7 The Breathe4T prototype intervention

The theory‐, evidence‐, and person‐based activities described above informed a draft prototype intervention, BREATHE4T. The intervention is a mobile‐friendly website providing a series of peer‐led training sessions to teach breathing exercises as well as providing advice and tips of how to integrate the techniques into daily living. The intervention guides users through the rationale behind BRT using a combination of videos from peers and physiotherapists. The main dashboard includes other optional features such as diaries (planning, reminders, and self‐monitoring tools), a progress chart and a frequently asked question section addressing common concerns. Users are able to work their way through training sessions at their own pace with prompts to move on once comfortable with each exercise. A shortcut is provided on the dashboard to access exercises that may be particuarly helpful to relieve asthma symptoms, alongside advice for how to use these in conjunction with an inhaler. A full overview of the intervention is described in Supporting Information: E‐Table 3.

## 3 DISCUSSION

### 3.1 A statement of principle findings

This paper describes the planning of a draft prototype intervention for adolescents with asthma following a theory‐, evidence‐, and Person‐Based Approach.^28^ The self‐guided intervention is based on a successful adult intervention (BREATHE) and aims to teach BRT. The approach enabled context‐specific issues to be identified that may be particuarly relevant for this age group, and for behavioural techniques to be implemented to address these. For example, the scoping review and interviews both highlighted embarrassment as a key issue toward using breathing exercises in public and this was considered when choosing intervention features by including techniques to increase self‐efficacy and relatedness, specifically by providing peer‐led training videos in relevant environments. Reminders were identified as an especially relevant feature, particularly if able to be tailored to an individual's preferences. The person‐based planning activities have led to a prototype intervention consisting of selected features intended to increase motivation and reduce potential barriers. Some preferences varied between individuals, and these issues were discussed with stakeholders to best meet key user needs. Future work will enable the prototype intervention to be further optimised using think‐aloud and retrospective interviews to explore a wider sample of user's reactions to the selected intervention content and target behaviours.

### 3.2 Strengths and weaknesses in relation to other studies, discussing important differences in results

This paper is the first in our knowledge to identify the key behavioural needs of adolescents with asthma that will be used to inform and develop a digital intervention that enables self‐management using breathing exercises. We provide a transparent description of the approach we took, increasing replicability. The method enabled a rich and detailed understanding of the intervention's target users and particularly understands their views of breathing exercises, an area that currently has very limited existing evidence for this age group.

Current evidence suggests that interventions including core integrated BCTs also tended to have higher quality ratings. Ramsey (2019) conducted a systematic evaluation of 23 existing asthma apps to identify the presence (or absence) of BCTs.^71^ More than half of the apps were identified as using less than four BCTs. The authors concluded that two existing interventions, KissmyAsthma and AsthmaMD used the most BCTs in addition to a high‐quality rating, assessed by MARS (Mobile App Rating Scale, used to assess quality of mHealth apps). KissmyAsthma study used a similar, theory‐, and evidence‐based approach and a recent pilot study shows promising results.^43^, ^47^ In the current study, our behavioural analysis used 30 BCTs, suggesting the Breathe4T intervention is in line with these current findings.

### 3.3 Strengths and weaknesses of the study

We followed a robust, theory‐, evidence‐, and person‐based process that has been extensively used to develop effective interventions.^33^ The intervention planning used extensive coparticipatory methods, working with participants and stakeholders, to maximise the likelihood of patients engaging with the intervention and thus its effectiveness.^26^ This will maximise the success of repurposing an effective adult intervention (BREATHE) ensuring it to be developmentally‐appropriate and likely to be acceptable and engaging for this patient group.^18^

The study included a small sample of local participants predominately based in Hampshire, South England. Although we aimed to recruit participants who varied across demographic measures and asthma severity as much as possible, our sample may not be fully representative of the target population. Specifically, the sample should not be considered representative of all views of all people with asthma, and further work should take care to ensure the intervention is acceptable and effective for people from a range of diverse socioeconomic and ethnic backgrounds. This will be addressed in the next stages of the study, as a nationwide feasibility study will significantly expand the number of participants and feature a more diverse population where potential differential benefits in subgroups can be explored.

### 3.4 Possible explanations and implications for clinicians and policymakers

This rigorous and user‐based method emphasises the possibility to engage an end user group throughout planning of an intervention, and particularly within a difficult‐to‐recruit teenage population. Adolescents have been receptive to the modality of an online intervention and it's increasingly acknowledged that digital interventions are relevant and accessible for this population. In addition, this study has informed a set of guiding principles to identify key design objectives (what the intervention is trying to achieve) and key features (what the intervention must do to achieve this) within this context (see Supporting Information:  E‐text 2).

### 3.5 Unanswered questions and future research

Our understanding of key, adolescent‐specific behavioural issues provide insight into the views and needs of this under‐served group. Along with the guiding principles informed by this study, these findings can be used to optimise interventions that address the needs of adolescents with asthma. However, care must be taken to ensure that key behavioural issues continue to be relevant within the behaviourally heterogeneous adolescent asthma population (such as those with milder asthma). Furthermore, the effectiveness and cost‐effectiveness of any potential interventions should be explored before implementation, and thus future research should include high quality randomised controlled trials.

## AUTHOR CONTRIBUTIONS

**Stephanie Easton**: Writing—original draft; writing—review and editing; methodology; formal analysis; project administration; investigation; validation; visualization; data curation; resources. **Ben Ainsworth**: Conceptualization; funding acquisition; writing—original draft; writing—review and editing; methodology; validation; formal analysis; supervision; project administration; visualization. **Mike Thomas**: Conceptualization; funding acquisition; methodology; writing—review and editing; validation; project administration. **Sue Latter**: Conceptualization; writing—review and editing; methodology; validation; formal analysis; funding acquisition; project administration; visualization. **Rebecca Knibb**: Funding acquisition; methodology; validation; writing—review and editing; formal analysis; project administration. **Amber Cook**: Methodology; writing—review and editing; project administration; resources; investigation; validation. **Sam Wilding**: Methodology; validation; writing—review and editing; project administration. **Michael Bahrami‐Hessari**: Resources; writing—review and editing; methodology; project administration; validation; investigation. **Erika Kennington**: Writing—review and editing; methodology; funding acquisition; project administration; resources; validation. **Denise Gibson**: Methodology; writing—review and editing; conceptualization; funding acquisition; project administration; validation. **Hannah Wilkins**: Methodology; writing—review and editing; validation; project administration. **Lucy Yardley**: Conceptualization; funding acquisition; methodology; validation; writing—review and editing; project administration; visualization. **Graham Roberts**: Conceptualization; funding acquisition; writing—review and editing; supervision; resources; project administration; formal analysis; validation; methodology; writing—original draft; visualization; investigation; data curation.

## CONFLICTS OF INTEREST

Mike Thomas, Lucy Yardley and Denise Gibson were coapplicants in the adult BREATHE study. The study was funded by NIHR RfPB. Erica Kennington is employed by Asthma Lung UK. The remaining authors declare no conflict of interest.

## Supporting information

Supplementary information.

## Data Availability

The data that support the findings of this study are available on request from the corresponding author. The data are not publicly available due to privacy or ethical restrictions.
