# Supplementary material for: Planning a digital intervention for adolescents with asthma (BREATHE4T): A theory‐, evidence‐ and Person‐Based Approach to identify key behavioural issues
Source: Pediatr Pulmonol. 2022 Sep 6;57(11):2589–602. doi: 10.1002/ppul.26099 (PMC9826240; doi:10.1002/ppul.26099)
Supplement: Supplementary file 1 — Supplementary information. [file PPUL-57-2589-s001.pdf]

Appendices

Planning a digital intervention for adolescents with asthma (BREATHE4T): a, theory-, evidence- and Person-Based Approach to identify key behavioural issues

Stephanie Easton BSc Hons[1,2], Ben Ainsworth PhD[3], Mike Thomas PhD[4], Sue Latter PhD[5], Rebecca Knibb PhD[6], Amber Cook BSc Hons[1], Sam Wilding PhD[7], Michael Bahrami-Hessari MSc[1], Erika Kennington PhD[8], Denise Gibson PhD[9], Hannah Wilkins BSc Hons[9], Lucy Yardley PhD[10], Graham Roberts DM[1,2*]

Affiliations:

1: NIHR Southampton Biomedical Research Centre, University Hospital Southampton NHS Foundation Trust, Southampton, UK. [1]

2: Human Development and Health, Faculty of Medicine, University of Southampton, Southampton, UK. [2]

3: Department of Psychology, Faculty of Humanities and Social Sciences, University of Bath, Bath, UK. [3]

4: Primary Care and Population Sciences, Faculty of Medicine, University of Southampton, Southampton, UK. [4]

5: School of Health Sciences, University of Southampton, Southampton, UK.

6: School of Psychology, College of Health and Life Sciences, University of Aston, Birmingham, UK.

7: Clinical Trials Unit, University Hospital Southampton, Southampton, UK.

8: Asthma + Lung UK, London, UK.

9: Physiotherapy department, University Hospital Southampton, Southampton, UK.

10: Centre for Clinical and Community Applications of Health Psychology, University of Southampton, UK.

Funding: 'This work was supported by National Institute of Health Research (NIHR) grant number [PB-PG-0817-20038].'

***Corresponding author:** Graham Roberts, Paediatric Allergy and Respiratory Medicine (Mailpoint 805), Southampton University Hospital NHS Foundation Trust, Tremona Road, Southampton SO16 6YD, United Kingdom. E-mail: g.c.roberts@soton.ac.uk. Tel. 02381206160.

Keywords: asthma, adolescence, self-management, breathing retraining, digital intervention

Abbreviated title: Planning a digital intervention for teens with asthma

Contents

E-Table 1, Key findings from scoping review ... 4

E-Table 2, Summary of barriers and facilitators from a scoping review of the literature ... 23

E-Text 1, Interview schedule ... 28

E-Table 3, Overview of the breathing retraining intervention for adolescents with asthma ... 32

E-Text 2, Guiding principles to develop the breathing retraining intervention ... 40

E-Table 1, Key findings from scoping review

Scoping review study summaries						
**Lead author**	**Year**	**Study type**	**Participants**	**Study aims**	**Intervention / method**	**Key findings**
**Abraham**	2019	Qualitative analysis	N=18 (8 children between 7-17yrs, taking 1 or more medications for chronic conditions, plus 10 parents)	To elicit views of medication and adherence apps	Participants interacted with and provided feedback on Medisafe adherence app<br>Semi-structured qualitative interviews<br>Thematic analysis	Nine themes emerged: ability to earn rewards for adherence;<br>having security functionality to protect user information;<br>customisable graphics and appealing colour schemes; medication-specific information; ease of accessibility to mobile devices; sharing child-friendly medication information; children's limited knowledge and involvement in medication management; and communication challenges with healthcare providers.
**Al-Sheyab**	2012	Cluster RCT	N=261 adolescent students	To determine the impact of a peer-led education program, developed in Australia, on health-related outcomes in high school students with asthma in Jordan.	Based on Triple A program – peer delivered<br>Measures at baseline/3months: Quality of life, self-efficacy and knowledge of asthma self-management	Improvements in all measures in intervention group – none in control group.<br>Clinically meaningful improvements in quality of life

**Barker**	2016	Single-arm intervention study	N=34 (7-16 yrs), clinical diagnosis of dysfunctional breathing, 21 with asthma, recruited from BreathWorks Clinic (UK)	To investigate the impact of breathing retraining for dysfunctional breathing	**All children received breathing retraining and education**<br>1-to-1, physiotherapist-led, with a parent (or active family member)<br>1.5 hr for first session, 1 hr subsequent<br>**Education:** explained condition, basic anatomy, mechanics of breathing, teaching of diaphragmatic breathing<br>**Teaching of diaphragmatic breathing** (to achieve nasal breathing, normal respiratory rate, tidal volume, feeling of control): taught in supine, then progressed through sitting and more demanding/functional positions<br>**Additional interventions taught where appropriate:** postural correction, thoracic mobility exercises, sniffs and vocal fricatives, relaxation, shortness of breath management<br>**Measures:**<br>PedsQL (parent proxy and child report versions); Nijmegen questionnaire	• 21 of 34 children referred to BreathWorks clinic during study had asthma<br>**First intervention to discharge:**<br>• 23 participants<br>• Median PedsQL child score improved (70.6 to 84.7)<br>• Median PedsQL parent score improved (58.6 to 82.6)<br>• Nijmegen Qu median symptom score decreased from 25 to 12<br>• All signif difs >0.0001<br>**After 6 months:**<br>• 13 completed questionnaires at all timepoints (T1, T2, T3)<br>• Improvements sustained at 6 months post-treatment.<br>21/23 children improved symptom score and QoL. Improvements sustained for those who completed follow-up.
**Bignall**	2015	RCT	N=33 (12-17rs, African-American, poorly	To test the feasibility, acceptability and preliminary	**Intervention group: 30 min breathing retraining plus education**<br>Two study visits, 1 month apart	• Intervention group had positive feedback: 66% preferred to practice to a CD

			controlled asthma)	efficacy of a school-based breathing retraining intervention in urban African-American adolescents with asthma	Education plus breathing-retraining<br>Breathing retraining:<br>• diaphragmatic breathing<br>• asthma-specific guided imagery<br>• progressive muscle relaxation<br><br>Relaxation/skills to control asthma and improve anxiety<br>Script plus CD to practice breathing at home<br>**Control: 30 min standard asthma education**<br>Pathophysiology of asthma<br>Standard symptom management techniques<br>Basic principles of mind-body connection related to asthma<br>Reminder to stay on medication routine<br>Educational handouts<br>**Primary outcomes:**<br>Asthma control (ACT), Asthma QoL (PedsQL), lung functioning (FEV1 and peak flow)<br>**Secondary outcomes:**<br>State anxiety (STAI - pre-post intervention), trait anxiety (over the 1-month period)	• Barriers to practicing at home: too busy, home life too chaotic<br>• **ACT:** Both groups improved in 1month period – but trend towards significant main effect for intervention group<br>• **PedsQL:** Not significant between groups, but both groups improved with time<br>• **STAI:** both groups reported decreased anxiety from pre-post intervention<br>• **FEV1:** no significant difference<br>• **Overall:** Asthma control improved more in intervention group – authors suggest even 1 session of breathing retraining might have potential to improve asthma control

					Qual feedback interview for acceptability/feasibility	
**Burbank**	2015	Single-arm feasibility trial	N=20 (12-17yrs, persistent asthma)	Feasibility of mobile asthma action plan (AAP) among adolescents	8 weeks mobile asthma action plan<br>Measures: usage rates, adolescent satisfaction, pre and post-ACT and self-efficacy scores	High satisfaction reported (93% of participants stated they preferred to control their asthma using the mobile AAP).<br>Participants with uncontrolled asthma showed significant increase in ACT post-intervention.<br>Significant improvement in asthma attack prevention domain post-intervention.<br>Nb. Most participants in this study had good asthma control at baseline.
**Carpenter**	2016	Qualitative interview study	N=20 and caregivers (12-16yrs, persistent asthma, owned a smartphone)	To evaluate strengths and weaknesses of 2 existing asthma apps (one targeted to children and one to adults)	2 apps were reviewed by participants<br><br>Initial impressions: participants had 10 minutes to explore the app then followed a 20-30 minute semi-structured interview, plus 2 closed questions (app usefulness, technology use)<br><br>Longitudinal: participants used apps at home for 1 week, followed by a 20-30 minute telephone interview	Asthma-related goals were mostly related to controlling/managing asthma.<br>Appointment and medication reminders, diaries, trigger and symptom tracking, charting, allergies and emergency plan, school reporting form and doctor reporting form were all deemed to be useful by most participants.<br>Facilitators included: improving asthma, getting off medications, reminder functions, convenience of documenting information, tracking progress over time, visualising asthma changing, monitoring own asthma severity, having emergency information in one place, sharing

						information, having various features, confidence, communication<br>Barriers included: lack of knowledge of triggers, interpersonal factors – attitudes and beliefs (already having knowledge of what to do in an emergency, competing demands, already doing a good job of managing asthma, not liking peak flows)
**Carpenter**	2016	Single-arm pilot study	N=25 (7-17yrs)	Using Tailored Videos to Teach Inhaler Technique to Children With Asthma	School-based, nurse-led study. School nurses assessed inhaler technique<br>Children then watched a tailored video that provided:<br>(1) step-by-step feedback on which steps (out of 8) they performed correctly<br>(2) praise for correctly-performed steps<br>(3) statements about why incorrectly-performed steps are important.<br><br>Nurses reassessed the child's inhaler technique immediately after watching the video and again 1 month later.	Inhaler technique was sustained 1-month after watching the videos.<br>In this pilot study, children were given the option of selecting the main character they wanted to watch.<br>96% selected a character that was concordant with their gender; girls selected female characters and boys selected male characters.<br>70% selected a main character that was racially concordant.<br>89% who chose a character that was discordant with their own race/ethnicity selected characters that were close to their own age.<br>Including peer role models as the videos' main characters may have increased children's motivation and self-efficacy.
**Chiang**	2009	RCT	N=48 children and parents	To examine the effectiveness of teaching a	Both groups received self-management program	Anxiety (state anxiety specifically) significantly lower in intervention group.

			with moderate-severe asthma	combination of self-management and relaxation-breathing techniques to children with moderate-to-severe asthma.	Intervention group also received 30 min of breathing retraining and a CD to practise at home<br>Measures: anxiety, peak flow, medication usage, perceived health status and asthma signs/symptoms	Other measures showed some differences between groups, but not significant.<br>Combination of self-management and breathing exercises has preliminary effects on children with moderate-severe asthma.
**Davis**	2018	Qualitative interviews for intervention development	N=20 (15-25yrs, 60% female, various levels of asthma control)	To inform development of an asthma self-management for asthmatic adolescents	Workbook (n=13) and face-to-face workshop (n=7)<br>(1) Phase 1: Lit review and workshop/workbook (20 ppts)<br>(2) Phase 2: Developed app based on phase 1 + created wire frames<br>(3) Phase 3: Think-aloud interview feedback (20-40minutes)<br>App naming via Facebook. A panel comprising members of the research team and Asthma Australia chose the winning name.<br>Activities:<br>A. Collage – identity as a young person with asthma<br>B. Quest life goals<br>C. Ideation of app content and features which would make the	Phase 1 results:<br>Asthma - adversary, something to be fought against, that restricts activities such as participation in sports and makes the person feel different and excluded. Valued features included emergency contacts, symptom tracking, and ways to connect socially.<br>Facilitators: Entertaining “look and feel”, reminders and notifications, rewards and feedback, off-line mode (to reduce battery use), content relevant to daily tasks, simple content, visual, and light on text, opportunity to connect with other young people with asthma, tracking of activity, medication, symptoms, and triggers, interactive guidance for asthma emergencies, work on different platforms (apple, android), support for goal setting, tutorial (can be skipped if not needed), mood diary, prefer swipe gesture to go

					app practical, useful and engaging (Head, Heart and Mind activity)<br>D. creation of screen designs 27 “wireframe” screens representing the core app functionalities were produced to conceptualize the prototype.	back, success stories, personalisation, informal tone, ease of use, simple login, pictures<br>Barriers: cost, adverts, separate back button, generic, difficult terminology or language, functionality elements such as ease of use and moving between screens.
**Davis**	2019	Pilot RCT	N=9 (15-25 years, doctor-diagnosed asthma)	To understand the user-experience and test the acceptance, engagement and effectiveness of the “Kiss myAsthma” goal-setting app for improving asthma self-management in a group of young people who piloted it for six weeks in their everyday life.	App use over 6 weeks<br>No comparison group	Clinically minimal important difference in quality of life.<br>High usability scores<br>Facilitators: flexibility, goal setting and reminders, logging/tracking asthma, sport and exercise goals, sharing objective evidence with HCPs.<br>Barriers: confusing features, not enough tips (too much focus on feelings), not all participants wanted to set goals.<br>Very small sample.
**Fedele**	2018	Qualitative development of an RCT	N=9 adolescent, caregiver dyads	To inform development of an asthma self-management app to be used in an RCT	Phase 1: advisory board to develop Aim2act intervention (mhealth tool)	Facilitators: engaging multimedia platform for generating videos, replacing text-based information with visual content wherever possible, and reducing the length of the videos to sustain attention.

**Grape**	2019	Program evaluation	N=259 (12-17yrs, persistent asthma)	Evaluate the acceptability of peer-led asthma self-management intervention in comparison to the program led by adult educators based on participant feedback.	Day camp by either peer-leader (N= 35, aged 16-20 years) or HCPs (control)	High acceptability<br>But no treatment group differences found<br>Comparable to when taught by HCPs<br>Relatability of peer-led participants was less robust – differences in socioeconomic status – though this did not diminish learning or satisfaction (but perhaps could be suggested that relatability is important for it to be even more effective).
**Hepworth**	2019	Service evaluation	N=169 (2-18yrs)<br>55 over 12yrs, mean = 10yrs<br>63% step 1-3<br>37% step 4-5		Attended individually tailored physiotherapy (including Buteyko breathing techniques)<br>Education: explanation of what normal breathing pattern was: nasal, diaphragmatic, quiet, slow and regular and how DB can affect asthma symptoms<br>- Diaphragmatic breathing inhaler technique was taught<br>- Subsequent physio treatments were tailored to the individuals needs and focused around Buteyko<br><br>Advised to practice Buteyko techniques 2x daily 10mins per time	Mean physio sessions = 6 (r = 2-20), with 2-4 weeks b/w appointments, 1 hour long<br>**ACT (primary outcome):**<br>Mean change in score - 69% signif improvement in ACT score.<br>Poor asthma control at 1st appointment – 20/30 on step 1-3 and 19/21 on step 4-5. Decreased to 8/30 and 8/21.<br><br>**NQ (dysfunctional breathing):**<br>Mean change in score was -9 (signif dif improvement)<br>**Overall:**<br>Physio (Buteyko + standard med management) – significantly improved asthma symptom scores and significantly decreased DB.

**Holley**	2016	Systematic review	N=16 studies<br>(11 qualitative<br>5 quantitative)	Review barriers and facilitators to self-management of asthma reported by adolescents using a narrative synthesis approach to integrate the findings.	Systematic review<br>Thematic analysis<br>N=1011 ppts, asthmatic, 12-18yrs	6 themes: Knowledge, Lifestyle, Beliefs and Attitudes, Relationships, Intrapersonal Characteristics, and Communication.<br>**Key findings:**<br>• Poor knowledge about asthma<br>• Non-adherence frequently caused by forgetting<br>• Setting routines helps self-management<br>• Erroneous beliefs about asthma and medication = disengage from treatment<br>• Difficult to control asthma without support from school<br>• Parents are key in reminding adolescents to take meds<br>• Many adolescents feel embarrassment about their asthma –<br>• particularly around friends/peers<br>• Difficulties communicating with HCPs
**Holley**	2018	Qualitative interviews	N=54<br>28 adolescents<br>12 parents<br>14 HCPS	Barriers and facilitators to self-management of asthma in adolescents	Barriers/facilitators into self-management (not just adherence to treatment)<br>Focus groups – 28 adolescents with asthma aged 12-18yrs; 14	10 themes: forgetting + routines, knowledge, embarrassment and confidence, communication with HCPs, triggers, support at school, apathy and taking responsibility.

					healthcare professionals and 12 parents<br>Thematic analysis and triangulated the views	Adolescents, parents and healthcare professionals raised similar barriers and facilitators to self-management.
**Kohut**	2016	Qualitative study	N=52 phonecalls	Qualitative analysis of iPeertoPeer	Content analysis of peer mentoring phone calls	Mentors shared experiences of illness impact<br>Mentors empathized and drew on personal experience to help mentees work through these concerns and associated guilt.<br>Discussed strategies that work for them to manage pain (coping skills), setting goals (both health related and not), as well as strategies that help with adhering to their treatment plan.
**Koster**	2015	Qualitative interviews	N=21 (12-16yrs, asthma)	To assess adolescent needs and preferences towards asthma support, with a focus on new media.	Two online focus groups and one face-to-face focus group<br>Questions concerned adherence behaviour in general and needs and preferences in adherence support with special focus on new media (e.g. mobile technology, social media and health games).	Themes: Adherence, role of parents, solutions to support medication use, new media as solution<br>Barriers: forgetting, lack of perceived need, gamification (more suited to younger)<br>Facilitators: reminders, peer support, easy-access smartphone app, sharing experiences with other teens, parental support
**Knibb**	2020	Systematic review	30 papers, data from 27 studies	The effectiveness of interventions to improve self-management for adolescents and young adults with allergic conditions	Narrative synthesis	E-Health interventions reported significant improvements for inhaler technique, adherence and quality of life.<br><br>Peer-led interventions - improvements were found for self-

						efficacy, school absenteeism and quality of life.<br><br>Small/pilot studies, some reported no effect sizes
**Masuda**	2013	Qualitative interviews	N=57 (12-15yrs, asthma)	To develop and evaluate an online peer mentorship program for youth with asthma and allergies	12-week online peer support participatory study for adolescents with asthma and allergies<br>Mixed-methods approach of interviews and surveys	Participants were satisfied with the opportunity to talk with others. Adolescents with asthma and severe allergies were able to post messages on the bulletin and send private e-mails and messages at any times to others in the group including the peer mentors. Participants were also satisfied with the mentorship they received from health promotion professionals during this program. The present study concludes that it was effective in engaging an age group that is difficult to reach in the program.
**Nightingale**	2017	Qualitative interviews	N=36 with chronic kidney disease (CKD)<br>19 individual interviews and 8 were joint – either with a parent or both parents	To inform development of a digital intervention.	Framework Analysis and behaviour change theories.<br>Semi-structured individual or focus group<br>interviews, depending on the individuals' preferences	Three key themes:<br>(1) Gaps in current online information and support<br>(2) Difficulties experienced by children with a long-term condition<br>(3) Suggestions for a digital care-management app
**Odom**	2016	Survey	N=12 (12-19yrs, asthma diagnosis)	To assess the ease of use and design of an AAP app.	Survey conducted to assist with development of an intervention	Barriers: tedious login process,<br>Facilitators: communicate with providers, portable, easy to use

**Panzera**	2013	Qualitative interviews	N = 18 teen dyads (13-19yrs, asthma and their caregivers)	To identify mechanisms for improving asthma self-management and characteristics for developing a digital intervention	36 semi-structured interviews (30-45mins)	Technology facilitators: ease of use, multifunctionality, portability, connecting to others, large memory capacity. Trusted information sources.<br>Preferences: a personal profile, information including symptoms, asthma condition, severity, status, inhaler use, triggers and daily activity. Reminders/alerts via text messages: some prefer before school, others afternoon. Recommendations for how to improve asthma, data about things that influence asthma (pollen count, weather), triggers and motivational and supportive messages, information and alerts shared with caregivers and HCPs. Facts and tips to prevent asthma. Videos (<10mins), visual tools to relay info to peers. Caregiver alerts that instruct teen to take med, call doc or calm down. Visual appeal, bright colours.<br>Barriers: unnecessary to have a social network site, strangers in the group, lack of a moderator, sharing personal information, security concerns.
**Peters**	2017	Qualitative participatory study	N=20 (15-24yrs, asthma)	Provide qualitative results of the	Theoretical thematic analysis	Overwhelming focus on the psychological experience of living with asthma

				psychological experience of asthma by YP and provide examples of how an asthma app can support this.		(unprompted/unanticipated by the researchers).<br>Key themes consistent with SDT<br>Asthma goals grouped in 4 themes: study, job/career, health/fitness/sport and spirituality/happiness/meaning.<br>Barriers to asthma goals: asthma and related illness (e.g. allergies), lack of money, lack of motivation.<br>Facilitators (support): family, friends, personal traits (determination), support for mental health/anxiety.<br><br>Preferred features: profile (medical history and treatment summary, medication list, customisable/personalised to user), reminders (medication alerts, find inhaler, located-based reminders), tracking (symptom diary, causes or trigger diary, asthma attack diary, mood diary, health over time graph, step count, overall log feedback, log report to show doctor), social (chat with others with asthma, Q&A forum, share personal experiences, share with friends, share story), emergency support (chat, automated chat, expert and how-to videos, list of specialists nearby, personalised asthma facts and stats, info to prevent attack), feedback and

						motivational features (support messages, daily motivation, competing).
**Ramsey**	2018	Qualitative interviews	N= (13-18yrs)	To obtain a deeper understanding of adolescents' general and health technology uses and their perceptions of how health technology may be beneficial in improving asthma self-management	Semi-structured interviews<br>Grounded theory	Themes:<br>Tracking asthma symptoms and medication, reminders, asthma knowledge.<br>A desire to customize technology to fit patient preferences and a desire to share collected data with medical providers.
**Rhee**	2011	RCT	N=112 (13-17yrs, asthma)	To evaluate the effectiveness of a peer-led asthma program compared to a program led by adults.	Day camp with group activities<br>12 peer leaders (10 females, 2 males, 16-20yrs).<br>Younger teens - two gender matched groups and a co-ed group<br>Older teens - three gender-mixed groups.<br>Power Breathing™ program was used for the campers' self-management training, 3 sessions (basic asthma education, psychosocial issues and asthma self-management skill).<br>Session lasted 45–60 minutes;	The intervention group reported more positive attitudes at 6-months and 9 months post-intervention than control.<br><br>Greater benefit to males and low income (higher improvement in attitude and QoL)<br>53.5% reported perceived emotional support from peer leaders and 51 % relied on emotional support from their leaders.<br>The majority of participants perceived peer leaders as good listeners, open, sensitive, and comfortable in confiding in them.<br>Thus, participants rated peer leaders

					Monthly phone follow-ups to provide continuous peer support.	high on knowledge, attitudes, personal skills, and perceived similarities. Further, all peer leaders reported enjoying their role as leaders.
**Roberts**	2016	Qualitative interview study	N=20 and caregivers (12-16yrs, persistent asthma, owned a smartphone) plus 3 friends	To identify adolescent, caregiver and friend preferences for using apps	Same method as Carpenter – plus adolescents were also asked to share study information with their friends who were invited for an interview	63% of adolescents reported wanting their friends involved in an asthma app
**Roberts**	2018	Carpenter above	N=20 (12-16yrs, persistent asthma, owned a smartphone)	To assess adolescent preferences and design recommendations for an asthma self-management app.	Thematic analysis for qual data<br>Quantitative questions analysed in SPSS	Facilitators: ease of use, visual appeal, customisation, functionality, tailored to informational needs, quiz, inspiring pictures, charts, colourful<br>Barriers: too basic, operating systems, graphics not good enough, difficult to use, too complex
**Roberts**	2019	Carpenter above	N=20 (12-16yrs, persistent asthma, owned a smartphone)	To gain feedback from adolescents with asthma on two existing asthma self-management apps and use their input to guide the development of a theory-based, engaging asthma self-management app	Classify features of two existing apps as predisposing, reinforcing and enabling use.<br><br>Precede-proceed model	Facilitators: sharing content easily with HCP, improving knowledge, progress tracking and symptom monitoring, rewards, triggers, diary, reminders, fun, emergency feature, feedback from the app

**Sage**	2017	Qualitative interviews	N=8 (11-18yrs, persistent asthma, smartphone)	To access the usability of a wireframe for an asthma app intended for use by asthmatic adolescents	Asked to evaluate 76 wireframes:<br>Visual aspects<br>Intended functionality<br>Areas for improvement<br>Expectations<br>Behavioural intentions	Facilitators: design, colours, "clean and professional", idea of an avatar, information on how medication works, how to avoid triggers, how to tell the difference between inhalers, how to tell when asthma is controlled, how to talk to doctor about medication, gamification, asthma control quiz – if short and accessible, customisation, quick to use "go more quickly", reminders for medication/doctors appointments, trigger alerts,<br>Barriers: rewards that don't translate to anything, bar charts, lack of customisation, labelling (specific graphics/icons, games
**Sezgin**	2018	Qualitative interviews	N=13 teens and caregivers (13-18years)<br>At least 1 chronic condition for >6 months	Which digital solutions may help to overcome the challenges and barriers to self-management	Individual sessions with the patient and the parent, followed by a joint interview session	10 themes identified relating to challenges, barriers and digital opportunities for teens with chronic conditions.
**Schneider**	2019	Qualitative interviews	N=20 (12-17yrs, asthma, mobile device)	To explore experience using asthma self-management app.	Self-management asthma app tested for trial period of 3 months<br><br>2 daily reminders to use app	Themes: app features, access, asthma management<br>Recommendations for improvement:<br>Barriers: outdated, too many reminders are annoying.<br>Facilitators: More visual, receiving training material in short videos, info on how to self-manage, general asthma info and how to use the app.

						Role models (celebs/athletes), HCPs, communication. Fun and entertaining elements.
**Shah**	2001	RCT	N=272 (students with recent wheeze, Australia)	To determine the effect of a peer led programme for asthma education on quality of life and related morbidity in adolescents with asthma.	The triple A peer education programme.<br>1) Student volunteers trained<br>2) Conducted ¾ health lessons for peers<br>3) Students developed and presented key messages learnt in the lessons to the year 7 students.	Effective in improving QoL and school absenteeism<br>Males were most affected by the intervention in the emotional domain and females were most affected in the activities domain.
**Stinson**	2016	Pilot RCT	N=30 (12-18yrs)<br><br>Juvenile Idiopathetic arthritis (JIA)	To examine the feasibility and acceptability of an online peer mentoring program (iPeer2Peer Program) for adolescents with JIA.	15 intervention<br>15 control (waiting list)<br>6 trained peer mentors (16-25yrs) with controlled symptoms<br>Participant mentor pairings connected ten times over 8 weeks using Skype video calls	Participants most enjoyed a) meeting someone with JIA who they could relate to, b) meeting someone older who has already experienced what they are going through (both JIA-related and non-JIA related), c) having someone to talk to, and d) getting information about JIA.<br>Participants reported satisfaction with the program and all reported that they would recommend it to their peers.<br>Engaged with programme.<br>Improvements in perceived ability to manage condition compared to controls.
**Vazquez-Ortiz**	2020	Systematic review	N=108 papers describing 106 studies	Review challenges and specific needs of adolescents	A descriptive synopsis with data tables was produced to summarize the literature	Five themes<br>a) Health-related quality of

			Young adults (11-15years)	and young adults with allergic conditions including measures of self-management and wellbeing.	Meta-synthesis approach	life—impairment was associated with poor disease control, psychosocial issues, adolescent-onset allergic disease and female sex<br>(b) Psychological factors—asthma and food allergy were associated with anxiety and depression, atopic dermatitis was associated with suicidal ideation, and that parental emotional support may be protective<br>(c) Adherence—suboptimal adherence was associated with older age, barriers to medication usage, poor symptom perception and failure to take responsibility, and positive factors were routines, simpler treatment regimes, better knowledge and perceptions about medications<br>(d) Self-management—facilitated by education, knowledge and a positive attitude<br>(e) Supportive relationships
**Waite-Jones**	2018	Qualitative Interviews	N=8 children and parents (10-18yrs, JIA)	To gain a deeper understanding of what peer-based social support means to young people with juvenile arthritis and ways in which it could be best provided.	Semi-structured interviews with young people and their carers<br>Focus groups with HCP's<br>IPA analysis	Sharing life experiences with similar others provides support through a sense of connectedness and improving confidence.<br>New kind of normality, understanding, relief and reassurance, shared learning,<br>Barriers: ambivalence towards peer contact (some prefer to be 'self-contained'), no-one size fits all - have

						to openly label your condition and risk being labelled

E-Table 2, Summary of barriers and facilitators from a scoping review of the literature

**Scoping Review:** Digital Interventions	**Scoping Review:** Peer-led Interventions	**Scoping Review:** Breathing Retraining	**Evidence from teams previous research (It's My Asthma)**

**Theme**	**Barriers**	**Facilitators**
User-specific issues and self-management	**Forgetting** asthma treatment (e.g. rushing before school, staying up late, out of routine or on holiday) (9, 17, 36-42)<br><br>**Too many reminders** (annoying) (42)<br><br>Feeling that **reminders are parent's responsibility** (37)<br><br>**Competing demands** (not wanting to interrupt leisure activities or unwilling to give up time with peers) (9, 36-39)<br><br>Treatment regimes conflict with **other priorities** (9, 17)<br><br>**Home life too busy or chaotic** to be able to practice breathing exercises (at home) (20)<br><br>**Older teens** or **those who have had asthma longer** have less preference to interact with other adolescents with asthma (36)<br><br>Feeling that they are **already doing a good job of managing their asthma** (36)<br><br>**Embarrassment (**of having asthma/using treatments in front of others) (9, 17, 35)	Being in a **daily routine** (9)<br><br>Having **cues** for remembering to take medication (9, 17, 20)<br><br>Appointment and medication **reminders** (9, 17, 36-45)<br><br>Ability to **customise/schedule** reminders (38, 40, 42)<br><br>Receiving **reminders from parents** or encouragement to take meds (9, 17)<br><br>Feeling **confident to take more responsibility** of their asthma/health condition (17, 36)<br><br>An opportunity to be **self-reliant** (36)<br><br>Empowering parents/caregivers to support their adolescent (23, 37, 42)<br><br>Parents can have a supportive role in breathing retraining (57)<br><br>Transferring responsibility from early adolescence (23)<br><br>Growing up and **deciding to take responsibility** (17)<br><br>**Acceptance** of having asthma (36, 39, 42)

Theme	Barriers	Facilitators
	Feeling restricted by asthma or unable to achieve personal goals (39)<br><br>Greater **anxiety** (23)<br><br>Negative beliefs, attitudes and perceptions towards asthma (9, 17, 23)<br><br>Lack of motivation (9, 17, 43)<br><br>Lack of social support and communication (9, 17, 35)	High self-efficacy (23)<br><br>Feeling in control of asthma symptoms (40)<br><br>**Goal setting** (39, 43, 47)<br><br>Taking a **calm approach** towards asthma (17)<br><br>To feel less limited in daily activities and to be able to achieve goals (36-39, 43)<br><br>Achieving or maintain a sense of normalcy (37-39, 43)<br><br>Support from peers (48)<br><br>Avoiding hospitalisation (48)<br><br>Smartphone is always carried and available (37)
Asthma content, education and training needs	Not having enough information and understanding about asthma/inadequate asthma knowledge (17, 36, 38)<br><br>**Lack of knowledge** about condition, symptoms, triggers (23, 36, 38, 40-43, 45, 49)<br><br>Lack of understanding about the severity and risks involved with asthma (23)<br><br>Intervention **not as replacement information and advice from HCP's** – prefer to be promoted as a supplement (44, 49)<br><br>Drawing attention to asthma (46, 48)	Being given **new ways** to control asthma (17)<br><br>Able to self-judge and monitor asthma changes and severity (36, 38, 40, 43, 49)<br><br>To reduce the need for appointments with HCP's (49)<br><br>Greater **understanding of consequences of condition** (23)<br><br>Credible and trusted source of information (38, 44, 45)<br><br>Knowledgeable social networks (peers, family, friends, school) (23)<br><br>Training facilitated by **older adolescent peers** (52, 54, 55)

Theme	Barriers	Facilitators
	Peer mentors not relatable; differences in socioeconomic status and race (50)<br><br>Supportive language (39)	Young people **prefer advice from peers** and change is more likely to occur if someone they can relate to or they perceive as a role model delivers the message (52, 54, 55)<br><br>Children preferred to learn from someone who was **demographically similar to them**. When given the choice of character in a peer-led video, most participants chose a character that was concordant with their gender and race (56)<br><br>**Peer video testimonials** of asthma experiences (48)<br><br>Wanting to interact/hear experiences of others who have asthma/same disease (relatedness) (36-38, 42-44, 46, 48, 52, 53)<br><br>An intervention that improves asthma control and allows users to **gain more control over goals** and lifestyle and physical activity (36, 38, 39, 42)<br><br>HCPs to share info, experience, advice and encouragement (39, 43, 45, 48, 49)<br><br>Information about communicating with HCP's – users should be **encouraged to contact HCP's for unanswered questions or concerns** (44)<br><br>Peer role models as the videos' main characters may have increased children's motivation and self-efficacy (36)<br><br>**Sex-matched** peer trainers (55)<br><br>Participants showed a preference to practice breathing exercises to a CD rather than alone (20, 57)

Theme	Barriers	Facilitators
		Breathing exercises enabled children to feel relaxed, calm and fall asleep easily at night (57)<br><br>Patients felt the breathing retraining intervention improved their psychosocial functioning. Trait anxiety (STAI) improved 1-month post breathing retraining and asthma education (20)<br><br>Asthma control improved in intervention group, compared to group receiving standard asthma education (20)<br><br>Adolescents practiced their breathing exercises when they felt asthma symptoms (breathlessness, wheezing), or to relax and calm down (20)<br><br>Emergency information (36, 43, 48)
Features and design preferences of digital interventions	Too many reminders are 'annoying' (42)<br><br>Inspirational messages that are **too repetitive** (42)<br><br>Too much information on one page **'cluttered/clumped'** (43, 45)<br><br>**Outdated** app design (42)<br><br>**Not understanding how to use the app** (43, 45)<br><br>Difficulty moving between screens (43)<br><br>**Tedious login process** (42, 58)<br><br>**Forgetting login** details and passwords (42)<br><br>Quiz too long (41)	Information that is **easily accessible** and shareable (with HCP's, parents, school etc.) (36, 37, 41, 42)<br><br>**Clean, professional, organised** (41)<br><br>Able to gain **feedback from providers** about asthma self-management (40, 45, 71)<br><br>Use of inspirational/**motivational messages** (42)<br><br>Ability to **earn rewards** for adherence (51)<br><br>Education and training in the format of **video tutorials**/picture explanations to aid understanding or to learn skills (36, 37, 40, 42, 43, 45, 48, 52)<br><br>**Length of videos kept short** to sustain attention (38, 48, 59)

Theme	Barriers	Facilitators
	Child-like (45)<br><br>Rewards that don't translate into anything (41)<br><br>Poor accessibility (e.g. Wifi, devices) (42, 45)<br><br>Too many features (45)	Use of **visual aids** such as colour, pictures, graphs and charts (41, 43, 45)<br><br>**Replacing text-based information with visual content**, wherever possible (59)<br><br>**Ability to customise, personalise settings and add own information** (37, 42, 44, 45, 59, 71)<br><br>**Ease of use** (43, 51, 58) - increased engagement with the app and facilitated continual use (45)<br><br>**App customisations** that fit with current schema, apps and technology that they are already familiar with (45)<br><br>Security functionality to **protect user information** (51)<br><br>Tracking: able to **visually track progress** over time (36, 45), triggers and symptom tracking and monitoring (36, 38, 40, 45)<br><br>**Self-check quiz** as engaging way to aid understanding (45)

E-Text 1, Interview schedule

About your asthma:

Can you tell me a bit about you and your asthma?

Is there anything that your asthma prevents you from doing?

Is there anything you'd like to be able to do that you think your asthma stops you from doing?

Breathing Exercises *[SHOW VIDEO FROM ADULT WEBSITE]***:**

Have you ever had any experience of using breathing exercises?

IF YES:

- Can you tell me about how you think breathing exercises may help?
- Can you tell me what you think the benefits of breathing exercises would be?
- Can you tell me any concerns you might have about breathing exercises?

IF NO:

- Can you tell me what you think about this idea?
- Can you tell me if you think they could be helpful for you?
- Can you tell me any concerns you would have about doing these?

[short explanation of breathing exercises]

If you were to practice some breathing exercises, can you tell me a bit about when and where you might practice them?

Can you tell me a bit about what you like to do to relax?

Can you tell me about times that you have found it difficult to control your asthma?

Are there any particular places/situations in which you find it hard to keep your asthma under control?

Breathing exercises can be used to help you to calm down if you are having an asthma attack.

Can you tell me about how you would feel to do some breathing exercises?

- What might help you / what would make it more difficult?
- Any situations that you'd feel it would be difficult?
- How would you feel using these breathing exercises around your friends?
- Are there any kind of situations that you think other people your age might find difficult about doing breathing exercises?

[SHOW VIDEOS OF BREATHING GIFS]

The website will teach you how to use each breathing technique. One of the techniques would be to help you slow your breathing down.

- Can you tell me what you think about these?
- Can you tell me any of your own ideas about these could look?
- Is there anything you particularly like about these?
- Is there anything you particularly don't like about these?

Design:

- If you're thinking about the way a website looks, are there any things you particularly like?
- Can you tell me a bit about websites that you like and why you like them?
- Can you tell me a bit about websites that you like and why you don't like them?
- Are there any particular features on a website that you like/dislike?

The website will give information about asthma and how to perform breathing exercises.

How would you want to see this information on the website?

- Can you tell me why you like that idea?
  - For example, we are currently thinking about videos or text..?
- Would you want to read the information? Why / why not?
- How else might you like to see the information?

Videos:

Some teenagers have told us the adult website has too much text and that they would prefer to watch short videos.

- Do you agree? Can you tell me more?

Can you tell me how you think these videos would best help you to learn the breathing techniques?

Either show video again or 'thinking about the video you watched':

- What do you think of this video?
- Can you tell me how you might improve it?
- Likes/dislikes
- Why?

Can you tell me a bit about the type of people you might like to deliver the training in the videos?

- Examples to suggest
    - Doctors/nurses
    - Older kids with asthma
    - Adults with asthma
    - Peers or alone?
    - Physio?

Can you tell me about anybody you particularly wouldn't like to deliver the training in the videos?

Where would you like to see these videos set?

- Backgrounds / locations

Features and engagement:

Can you tell me what might make you want to keep using the website?

Can you tell me if there is anything that may stop you wanting to carry on using the website?

Do you think rewards are a good or a bad thing?

- Can you tell me how you think rewards may be able to fit into this website?
- Would you like rewards to fit into this website?

Can you tell me your thoughts about a progress chart?

What are your thoughts about reminders/notifications?

- Emails or texts?
- What are your thoughts about receiving text reminders?
- How often would you want to receive motivational texts?

Goal setting

Use of website/using website at all

Can you tell me about how you might want your parents to use the website with you?

- Why / why not?

If you are answering questionnaires online:

Can you tell me your thoughts about answering questionnaires online?

- Can you tell me about anything you find unhelpful about answering questionnaires online?
- Can you tell me anything you might find helpful when answering questionnaires online?

Anything to add / we haven’t covered?

E-Table 3, Overview of the breathing retraining intervention for adolescents with asthma

<table>
<tr><th>Content</th><th>Description</th></tr>
<tr><td>Settings
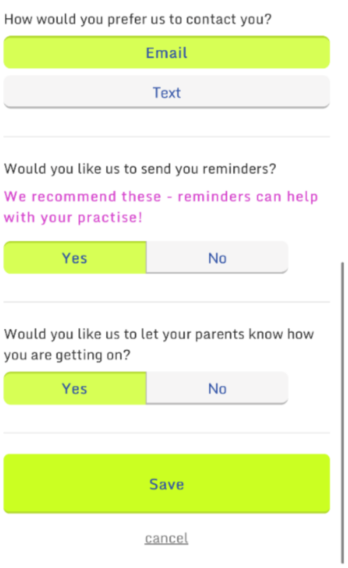</td><td>Personalisation:
- Contact preferences *email/text*
- Reminders *on/off*
- Update parents *on/off*</td></tr>
<tr><td>What is breathing retraining?
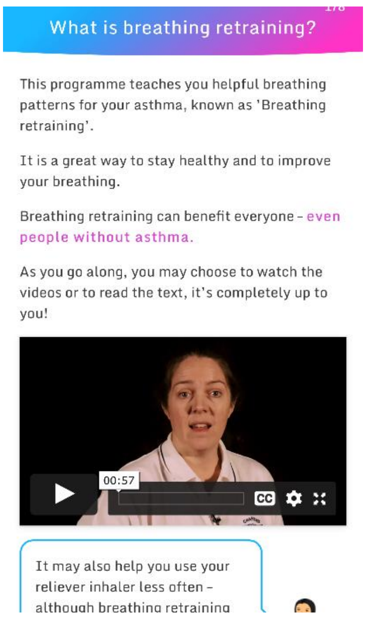</td><td>Video of physiotherapist describing the benefits of breathing retraining

Advice to use alongside medication</td></tr>
</table>

<table>
<tr>
<td>My breathing
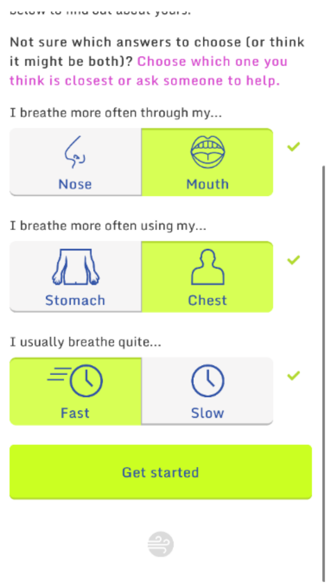
</td>
<td>Self-check quiz to reflect on current breathing techniques (mouth/nose, chest/stomach, fast/slow)</td>
</tr>
<tr>
<td>How it works
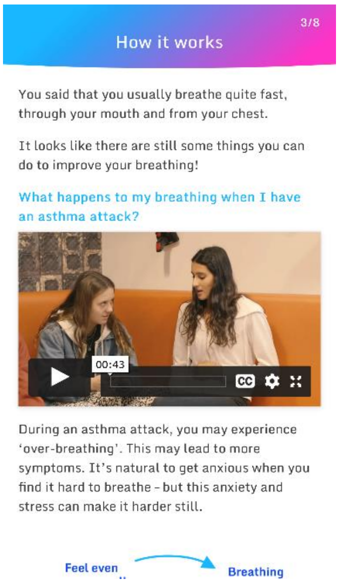
</td>
<td>Feedback on current breathing techniques
Information and peer video scenario of what happens during an asthma attack
Hyperventilation cycle
Information and peer video scenario of using breathing exercises to control breathing during an asthma attack</td>
</tr>
</table>

<table>
<tr>
<td>How does asthma affect breathing

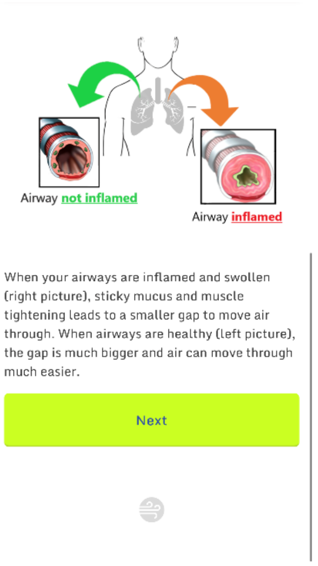

</td>
<td>Information and video describing how asthma affects the airways

Diagram and labels to compare airways with and without asthma</td>
</tr>
<tr>
<td>How breathing retraining helps

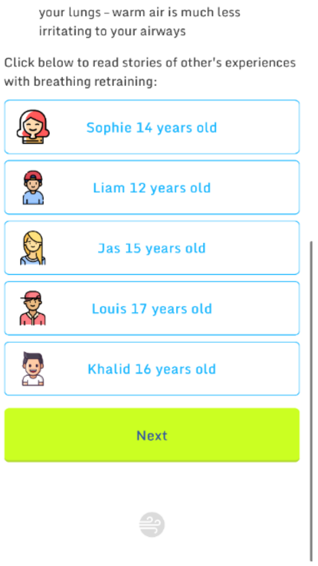

</td>
<td>Physiotherapist video describing the benefits of breathing exercises

5 short peer stories sharing experiences of breathing retraining</td>
</tr>
</table>

<table>
<tr>
<td>What exercises should I use, and when?

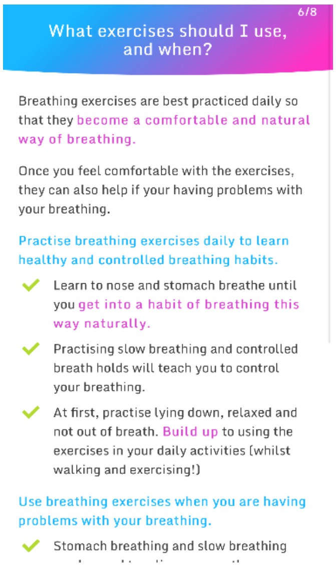
</td>
<td>Information to differentiate between exercises that build healthy breathing habits and those to use when having breathing problems.

Encouragement to build exercises into daily life.</td>
</tr>
<tr>
<td>How does Breathe4T work

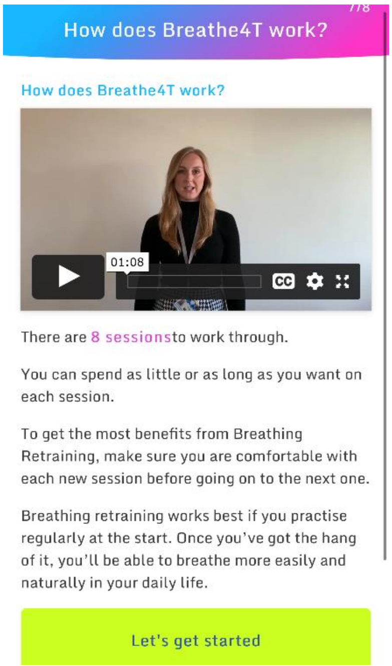
</td>
<td>Encourage practise before moving onto subsequent sessions.</td>
</tr>
</table>

<table>
<tr><td>Top tips
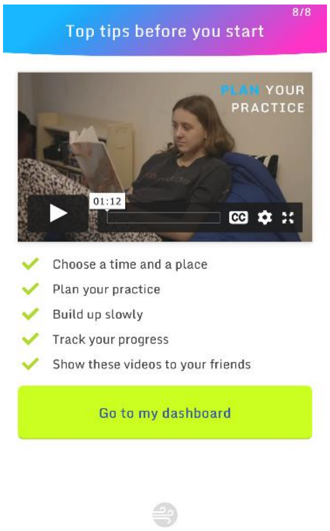
</td><td>Peer video with various tips for practising breathing retraining.</td></tr>
<tr><td>Dashboard: My inhaler use
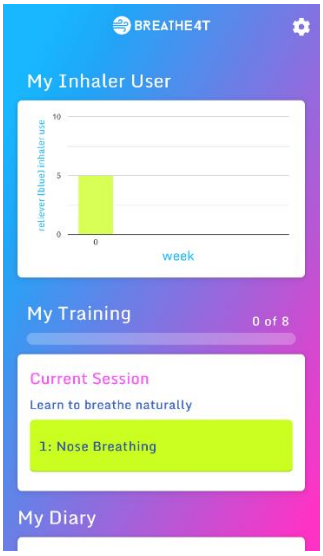
</td><td>Progress chart to monitor inhaler use over time</td></tr>
</table>

<table>
<tr>
<td>
Dashboard: My training

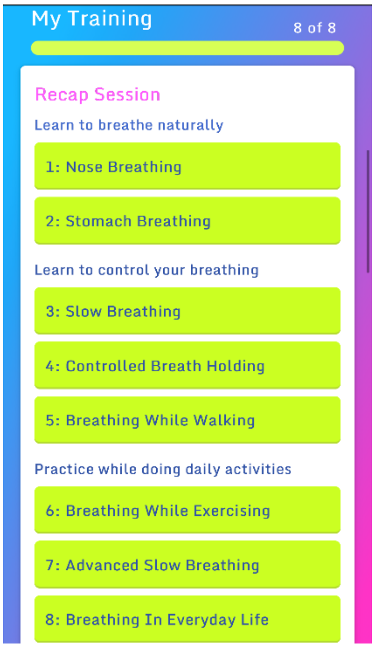

Slow breathing session example:

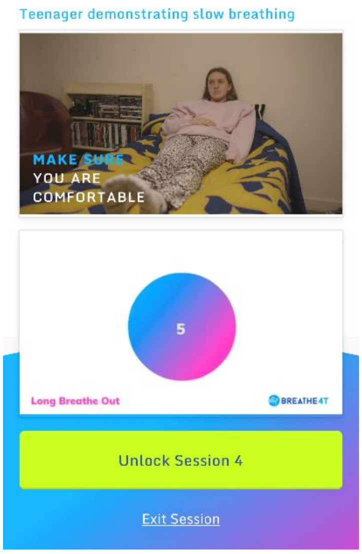

</td>
<td>
Breathing exercise training sessions. All include peer demonstration videos, step-by-step instructions and the rationale for the exercise:

- Nose breathing
- Stomach breathing
- Slow breathing
    - Animation/GIF to breathe in time with
- Controlled breath holding
- Advanced slow breathing
- Breathing whilst walking
- Breathing during exercise
- Breathing during daily life
</td>
</tr>
</table>

<table>
<tr>
<td>Dashboard: My diary
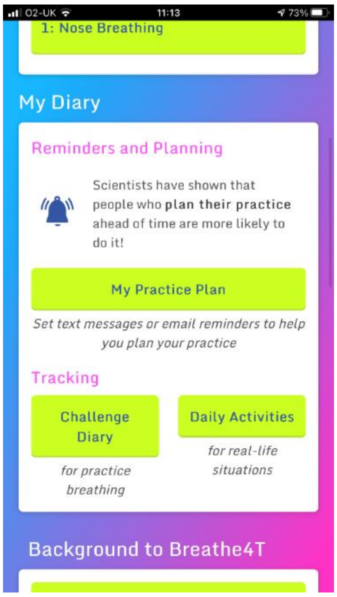
</td>
<td>Reminders and planning
Select a day/time/personal note to plan practice
Option to set a text/email reminders
Tracking
Challenge diary
To log the time spent doing the practice exercises
Daily activities
To log situations that breathing exercises have been used and to rate confidence level using smiley emojis</td>
</tr>
<tr>
<td>Dashboard: Having breathing problems?
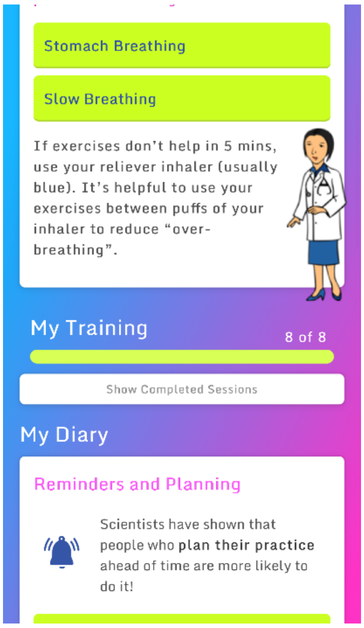
</td>
<td>Quick-links to rescue techniques that can be used to relieve asthma symptoms.
Advice on how to use the exercises alongside an inhaler.</td>
</tr>
</table>

<table>
<tr>
<td>Dashboard: FAQs
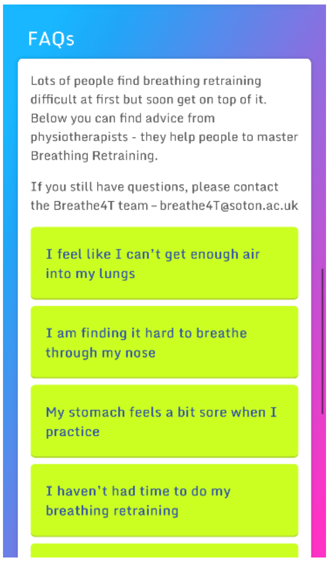
</td>
<td>Advice from physiotherapists about common issues relating to breathing retraining, including stomach soreness, not having time to fit it in and embarrassment of using techniques around friends without asthma.</td>
</tr>
</table>

E-Text 2, Guiding principles to develop the breathing retraining intervention

Purpose

To present the context-specific design needs and challenges for this target population and to outline the intervention features needed to address these.

Methods

Informed by evidence, theory and stakeholder input, the intervention objectives were outlined in terms of behaviour and outcomes. The relevant aspects of the target users and their context were listed, alongside the key behavioural issues, needs and challenges that the intervention should address. Based on this, the guiding principles were drafted. The intervention design objectives were presented alongside the key features needed to address them. The guiding principles were iteratively updated throughout intervention planning and optimisation.

Results

The objective of the intervention is to improve the quality of life of adolescents with asthma by supporting illness self-management using breathing retraining. Our scoping review identified that intervention users are likely to have uncontrolled symptoms, impeded by low adherence. Adolescents may also lack the knowledge, competence or motivation to take responsibility and self-manage their condition using non-pharmacological techniques. Therefore, an intervention design objective included to support users to gain autonomy around their asthma management. Another was to provide users with the competency to perform breathing retraining exercises and to persuade them that a combination of this approach, alongside their medication would be optimal to controlling their asthma. This required addressing any pre-existing concerns, misconceptions and beliefs and providing both accessible, yet trustworthy information about how to implement breathing exercises and reduce anxiety when experiencing asthma symptoms. The final design objective was to ensure content was engaging and accessible by reducing reading burden, providing choice and ensuring it was age-appropriate and in-line with current technology. Involving adolescents in the development process would help this to be achieved.

Intervention design objectives	Key features
To support adolescents to gain autonomy around their asthma management	• Use peer modelling to build adolescents self-efficacy for the target behaviours (e.g. short, peer-led content on how to practice breathing retraining exercises in relatable scenarios).<br>• Ensure adolescents can gain an appropriate understanding of their asthma and the rationale of breathing exercises.<br>• Interactive content of goal setting, planning/habit-forming routines and self-monitoring using visual aids, charts and graphs.<br>• Reminders and notifications that can be scheduled and customised to individual preferences.<br>• Persuade users that regular practice will lead to breathing exercises being performed subtly, reducing feelings of embarrassment in public.<br>• Provide guidance for how breathing exercises can be used, built upon and utilised when having problems with breathing (asthma symptoms).
To increase competency and persuade users that a combination of both pharmacological and non-pharmacological means (breathing retraining) is optimal	• Address concerns, misconceptions and erroneous beliefs about asthma and treatment.<br>• Provide quick-access information on how to implement breathing exercises and reduce anxiety during an emergency.<br>• Provide credible and trustworthy asthma information (including information about symptoms and triggers).<br>• Use self-check quizzes to provide feedback on current breathing technique.
To provide engaging and accessible intervention content	• Reduce reading burden by keeping information relevant, the amount of text per page to a minimum and by replacing text-based information with videos wherever possible.<br>• Provide choice and customisation wherever possible.<br>• Ensure the intervention is age appropriate, intuitive, in line with familiar technology and is mobile-friendly.<br>• Involve both adolescents and health care professionals in the development and design of the intervention.
